# Utilizing Machine Learning to Identify Gut Metabolites Associated With Alzheimer’s Disease

**DOI:** 10.1093/geroni/igaf122.1617

**Published:** 2025-12-31

**Authors:** Joshua Chuah

**Affiliations:** Union College, Schenectady, New York, United States

## Abstract

The prevalence of Alzheimer’s Disease (AD), which encompasses the vast majority of dementia cases, increases drastically in adults age 65 or older. In recent years, researchers have investigated the physiological relationships between the gut microbiome and the progression of AD. Furthermore, the early diagnosis of AD is important to improve patient outcomes. As such, the goal of this study is to develop a machine-learning based diagnostic test using gut metabolite data in order to facilitate earlier diagnosis of AD. Publicly available gut metabolite data was utilized, which included 104 gut-microbiome associated metabolites. These metabolites were measured from serum samples collected from 108 early mildly cognitively impaired (EMCI) and 145 AD patients. From these 104 metabolites, recursive feature elimination (RFE) feature selection identified 14 metabolites as being highly predictive between EMCI and AD status. A Logistic Regression classification model was trained using the data from these selected features and resulted in a 10-fold cross-validation accuracy of 91.34%. Moreover, robustness analysis of this classifier revealed that this classifier could still perform well with some missing values, maintaining above an 80% diagnostic threshold accuracy with approximately 13% of the data randomly re-sampled from a normal distribution (e.g., if missing values were randomly imputed). Overall, the predictive performance of this classifier indicates that it is both an effective and, more importantly, generalizable diagnostic test to distinguish between AD and EMCI individuals using gut metabolite measurements.

